# Development and evaluation of a participatory ergonomic intervention for the reduction of work-related musculoskeletal disorders among nurses

**DOI:** 10.3934/publichealth.2025063

**Published:** 2025-12-22

**Authors:** Guganesan Krishnanmoorthy, Rama Krishna, Faiz Baharudin, Mohd Amirul Asraf Shah Nizamuddin, Nik Hasmumthaj Nik Hassan, Mahmoud Danaee, Victor C W Hoe, Sanjay Rampal

**Affiliations:** 1 Department of Social and Preventive Medicine, Faculty of Medicine, Universiti Malaya, Kuala Lumpur, Malaysia; 2 Occupational Health Clinic, Universiti Malaya Medical Centre (UMMC), Kuala Lumpur, Malaysia; 3 Occupational Safety, Health and Environment Centre, Universiti Malaya, Kuala Lumpur, Malaysia; 4 Occupational Safety, Health and Environment Unit, Universiti Malaya Medical Centre, Kuala Lumpur, Malaysia; 5 Department of Civil, Environmental and Mining Engineering, University of Western Australia, Western Australia, Australia; 6 Department of Nursing, Universiti Malaya Medical Centre (UMMC), Kuala Lumpur, Malaysia; 7 Centre for Epidemiology and Evidence-based Practice, Department of Social and Preventive Medicine, Faculty of Medicine, Universiti Malaya, Kuala Lumpur, Malaysia

**Keywords:** musculoskeletal disorders, ergonomics, nurses, work performance, sick absenteeism, low back pain

## Abstract

Nurses are highly vulnerable to work-related musculoskeletal disorders (WMSD). This study evaluated the effects of a participatory ergonomic intervention on WMSD, sick absenteeism, and work performance among ward nurses. A multicomponent ergonomic training module, comprising educational ergonomics, health promotional activities, workstation adjustment, use of patient-assisted devices, and progressive muscle relaxation techniques, was developed based on a systematic review, focus group discussion, and discussion with ergonomic experts. A cluster quasi-experimental design was conducted, with nurses allocated into intervention (n = 45) and control (n = 30) groups, and follow-up across baseline, first, third-, and sixth-months post-intervention. The outcomes were assessed using the Nordic Musculoskeletal and Health and Work Performance Questionnaires. The intervention module was effective in reducing self-reported musculoskeletal symptoms among nurses across time, particularly neck pain [adjusted odds ratio (AOR) = 0.42, 95% CI 0.21–0.87], low back pain (AOR = 0.45, 95% CI 0.21–0.97), and shoulder pain (AOR = 0.38, 95% CI 0.17–0.88). However, the effects on relative absenteeism and relative presenteeism were only observed following adjusted analyses. While the ergonomic intervention was effective in reducing specific WMSDs among ward nurses, more research is required to delineate its potential application as a long-term strategy for addressing sick absenteeism and work performance.

## 1. Introduction

Work-related musculoskeletal disorders (MSDs) encompass various degenerative and inflammatory conditions affecting the skeletal system and locomotion apparatus, thereby leading to soreness, pain, and physical discomfort [1]. Apart from the direct effects on physical health and well-being, WMSDs negatively impact healthcare workers by increasing the risk of sick leaves, leading to poor work performance [2],[3].

Nurses are highly vulnerable to WMSDs as a result of multifactorial events, particularly their involvement in physically challenging activities such as patient mobilization, transfer between positions, and routine official duties [4]–[6]. Physical load and work posture [7], psychosocial factors such as the presence of psychosomatic symptoms and personality, work tasks, and work organization are also pertinent predictors of WMSDs among nurses [5].

Healthcare workers, especially nurses, face a high burden of WMSD in Malaysia, with a prevalence ranging from 55% to 80% [8],[9]. Approximately 8 in every 10 nurses in Klang Valley suffered from WMSD symptoms within the last year [8]. Given the adverse consequences of WMSDs, there is growing interest in developing effective workplace interventions in addressing these public health concerns. Accumulated evidence from the literature suggests that musculoskeletal injuries and the associated functional disabilities could be ameliorated by deploying occupation-specific interventions and rehabilitation options [10],[11].

Ergonomic interventions, particularly participatory ergonomics (PE), are well-documented preventive and treatment modalities for WMSDs [12]. PE integrates large-scale interventions that are carried out at organizational and system levels with small-scale interventions where workers are equipped with knowledge in tackling work-related ergonomic issues [13]. Furthermore, PE entails the active involvement of workers in controlling and planning a substantial amount of their work activities, combined with workplace changes, ergonomic knowledge, and procedures to improve working conditions, safety, and productivity [12].

Recent studies have shown the effectiveness of single and multicomponent ergonomic interventions in addressing musculoskeletal pain [10],[14]. Meanwhile, other researchers reported limited success of ergonomics interventions in ameliorating WMSDs, work performance, and sick absenteeism [13]. Differences in the intervention design, study samples, data collection tools, settings, and research design may contribute to the discrepancies in results. Overall, limited information is available on the appropriate and effective ergonomics interventions for either preventing or managing WMSDs and their related consequences among nurses.

In Malaysia's healthcare context, despite the high prevalence of WMSD, evidence-based data are lacking to support the development and implementation of ergonomic interventions to address the problem [4]. Given the multifactorial nature of WMSD among nurses, a multifaceted approach is necessary to effectively mitigate the determinants and the development of WMSD in Malaysia's healthcare settings. This study aims to evaluate the effects of a PE ergonomic intervention in addressing WMSD, sick absenteeism, and work performance among ward nurses at the Universiti Malaya Medical Centre, Malaysia.

## 2. Materials and methods

### 2.1. Study design and ethical approval

A cluster quasi-experimental design was conducted by recruiting nurses from the Universiti Malaya Medical Centre (UMMC) and allocating them either to the intervention or control group without using a randomization method. The research approach was conducted using a prospective six-month parallel two-armed design. Approval for the study was granted by the Medical Research Ethical Committee of Universiti Malaya Medical Centre (MREC ID No: 20221019–11632) and National Medical Research Register (NMRR ID–23–0184–F44). The study duration was from September 2023 to July 2024. Clinical trial registration was not performed for this study.

### 2.2. Study population and recruitment

All participating nurses were recruited from the UMMC. All the nurses provided both signed the informed consent form before they were recruited in the study. An information sheet and consent form describing the study were sent to the staff members. Interested individuals indicated their readiness to participate either via email or contact numbers provided in the information sheet. Thereafter, clusters were created before assessing if nurses fulfilled the inclusion criteria. Nurses were enrolled from any of the three wards (11U, 12U, or 13U) in UMMC, which are the medical units identified with a higher risk of WMSDs, as reported in a previous study [6]. In the study, nurses reported MSD prevalence of 86.9%, with no significant difference between the wards, thereby indicating a similar risk level and susceptibility to MSD.

The wards selected for the intervention group (12U and 13U) and the control group was the multi-interdisciplinary units in internal medicine wards (11U). This cluster allocation was performed purposively. Patients in the selected wards have similar diagnoses and treatment regimens, which often entail various levels of patient handling and transfer. Most patients admitted into these wards are physically incapacitated and scheduled for rehabilitation, palliative or medical procedures, reflecting the increased demands and workloads for nurses. Moreover, the specific nursing tasks and workloads in these wards are greater relative to other wards in UMMC, which range from routine functions like catheterization and dispensing medication to more intensive job demands like assisting physicians during medical procedures and patient transfer pre- and post-operatively. These functions and the lack of supervisory support were identified as risk factors for WMSDs among nurses in UMMC [6]. Thus, both psychosocial and organizational factors contribute to the nurses' vulnerability to WMSDs in the selected wards.

### 2.3. Sample size determination

The required sample size was computed using G*power 3.1 software upon selecting vital parameters from relevant studies [14],[15]. The parameter estimates were T-tests and bivariate regression with 2 groups and between slopes as the statistical test, precision level of 0.05, study power of 80%, allocation ratio of 1:1, slope value of 0.60, standard deviation residual of 2.0, and the corresponding standard deviations in the intervention and control group at the six-month follow-up point. The sample size obtained from the referenced studies [14],[15] for each group was 30 and 27, respectively. Hence, a larger sample size of 30 was selected for the study. Upon adjusting for a 10% dropout rate, the total number of nurses required in each group was increased to 33.

### 2.4. Inclusion and exclusion criteria

The inclusion criteria entailed nurses who were actively working in UMMC for at least the last 6 months before the time of enrolment, suffered from one or more WMSD, and had no history of trauma, surgery, or pregnancy. Meanwhile, nurses who were pregnant, were apparently healthy, had worked in the UMMC for less than 6 months, and those who underwent any kind of trauma or surgery on the body parts susceptible to WMSD were not considered for inclusion.

### 2.5. Sampling method

Three medical-based wards were selected as clusters (11U, 12U, and 13U) based on prior information and reports at UMMC, suggesting high risks of WMSD among nurses and healthcare professionals in these units. The clusters were allocated, with two wards (12U and 13U) designed specifically for the intervention group, and the third ward (11U) designed for the control group. Upon fulfilling the inclusion criteria, nurses from 12U and 13U were allocated to the intervention group, while those from 11U were selected as the control group. Allocation concealment and blinding were not performed in this study since the experimental protocol was designed to implement an ergonomic interventional module.

### 2.6. Contamination control

The main source of potential contamination in this study was the possible exposure of the control group to the ergonomic intervention, particularly among nurse staff sharing shifts. Despite not performing cluster randomization, contamination was mitigated by selecting specific wards as intervention (12U and 13U) and control (11U) groups rather than sampling individual nurses from any ward. This strategy was used given the settings at the study hospital, where confining the intervention to only the treatment group would be challenging.

Another step taken to minimize contamination was by establishing detailed protocols for the intervention and control groups. Details on these measures are provided in the subsequent sections. Detailed information was provided in the intervention module, ensuring that participants understood the experimental procedures and intervention contents. They were also briefed to keep all information gleaned from the intervention private and avoid sharing such information with nurses from other wards. These measures assisted in restricting the control group from being exposed to the intervention elements either intentionally or unintentionally.

### 2.7. Study instruments

Two research instruments, which have been validated in the Malaysian education system, the Nordic Musculoskeletal Questionnaire (NMQ) and Health and Work Performance Questionnaire (HPQ), were used in this study to assess WMSDs, sick absenteeism, and work performance from baseline to six-month post-intervention. The rationale for using the NMQ stems from its capacity to gather reliable information on the onset, prevalence, and implications of musculoskeletal pain. It can also be administered via personal interview and self-completion [16]. The NMQ consists of 2 sections. Section 1 collects general information to identify areas of the body causing musculoskeletal pain, and section 2 gathers information on any accidents affecting each body area, functional impact at home and work (change of job or duties), duration of the problem, assessment by a health professional, and musculoskeletal problems in the last seven days [17]. The NMQ has been successfully validated in the Malaysian healthcare setting, demonstrating strong internal consistency [8],[18].

Meanwhile, the HPQ is a self-report tool for assessing job performance [19] that comprises two components: absolute and relative presenteeism. Absolute presenteeism indicates real performance, whereas relative presenteeism reflects performance in comparison to most workers' performance in the same profession [19].

The Malay version of the Health and Work Performance Questionnaire (HPQ) presented a Cronbach's alpha of 0.78, indicating acceptable reliability for assessing workplace performance [20]. A short form of the International Physical Activity Questionnaire (IPAQ) was used to assess their physical activity levels for baseline assessment, as well as participants' baseline vigorous, moderate, or walking activities based on the time and frequency spent in performing these activities. Since these events were not primary outcomes in this study, the short version of IPAQ was considered appropriate to record baseline data for these physical activities. Moreover, physical activities were not assessed during the follow-up periods as the experiment was performed only to assess the feasibility and effectiveness of the intervention.

### 2.8. Implementation of the participatory ergonomic training module

The PE training module was developed upon triangulating the findings from the systematic review and focus group discussion (FGD) sessions. The five main components in the intervention module comprised educational ergonomics, health promotion activities, workstation adjustment, usage of patient handling devices, and physical activities/exercise. These components were integrated to address both psychosocial and organizational risk factors for WMSD.

Each section of the PE intervention module was presented during the course by the principal investigator. Likewise, the sessions module was conducted at the Occupational Safety and Health department of the hospital, supervised by the principal researcher and a research assistant. The session module was performed either physically or online, depending on the circumstances during the study. Nurses were trained in each session, and they were provided guidelines on ergonomic risk assessment and a developed PE intervention module, namely Ergorealm. Details of the intervention module and contents are shown in Table 1, covering key aspects of participatory ergonomics such as worker co-designs of organizational changes, workstation adjustment, health promotional activities, and patient handling and assistive devices. The information was shared during the FGD and training sessions and supported with videos where necessary.

In addition to the components described in Table 1, the main exercise was progressive muscle relaxation (PMR), which was performed by ergonomically trained personnel. This was gleaned from the FGD findings and previous literature reflecting the effectiveness of PMR for addressing psychosocial risk factors contributing to WMSD in nurses [21]. Moreover, it is an effective, simple, and feasible technique that can be performed to ameliorate stress and anxiety, even during working hours and shift hours [22]. A combination of PMR and other ergonomic interventions has consistently resulted in enhanced efficacy, surpassing its standalone effects [22]. Detailed steps were video-recorded and distributed to the participants. A web link to the video was also provided to the participants in the intervention manual sent electronically using the Linktree application.

**Table 1.** Contents of Ergorealm and its domains, information provided, and method of administration.

Contents	Domains	Information provided	Method of administration
Educational ergonomics	• Knowledge of WMSD • Ergonomic principles • Progressive muscle relaxation	• Prevalence and ergonomic risk factors • Various manifestations of WMSD and body regions affected • Evidence-based information on PMR	• FGD • Training module
Health promotion activities	• Compliance with ergonomic principles • Ergonomic risk assessment	• Adopting neutral body postures • Reducing excessive muscular force • Keeping work items within easy reach • Working at proper heights • Reducing excessive repeated motions • Providing clearance and space for accessibility, muscle recovery activities • Maintaining a conducive working environment	• FGD • Training module
Workstation adjustment and training	• Nurses' roles and responsibilities • Functions of the intervention facilitators (i.e., ergonomics-trained personnel). • Ergonomic assessment and implementation.	• Preventative and protective measures (suggested solutions) to reduce the risk of ergonomic issues • Ensuring compliance with ergonomic training, addressing workplace stress • Possible consequences of not using patient handling/assisted equipment, • Preparing documentation sites • Creating adequate space for better workstation performance.	• FGD • Training module
Patient handling and assistive devices	• Patient-assisted devices during transfer, lifting, and repositioning. • Safety concerns and considerations before transferring patients	• Bed to a stretcher patient transfer • Bed to a wheelchair patient transfer • Slide boards transfer • Log-rolling technique • Moving patients either up in bed or side to side, and wheelchair repositioning	• FGD • Training module • Videos
Physical activities and exercise	• Progressive muscle relaxation	• Detailed information on the method, duration, and frequency of executing the physical activity	• Videos • Ergonomics-trained personnel • FGD

Note: FGD = Focus group discussion.

Three sessions of PMR were performed weekly for six months, equaling a total of 12 sessions/month. Participants were instructed to self-report two of the PMR sessions, while one session was to be self-recorded and uploaded as a video into the Google form via the link provided. Uploaded videos were evaluated to ascertain if the participant performed the PMR correctly. Participants' privacy and confidentiality were maintained. Supervisors in the ward were primarily responsible for ensuring participant compliance by viewing the videos of nursing positions ergonomically and the PMR exercise.

The effectiveness of the intervention module was evaluated at one month, three months, and six months post-intervention. The assessment protocol commenced with a baseline measurement, followed by the PE intervention module for WMSD. During each session, participants were allocated enough time to complete the study instruments. Two assessments (outcome assessment and compliance assessment) were conducted in the intervention group. As for the control group, the participants were given an educational brochure from the Department of Occupational Safety and Health (2017). The educational material is a usual care provided for all ward nurses actively working in the hospital. Only one outcome assessment was performed in the control group.

### 2.9. Outcomes assessment and operationalization

An online Google form was used to collect participant-reported information and assess outcome measures. The primary outcome was self-reported WMSD, with sick absenteeism and work performance the secondary outcomes. The self-reporting instrument was designed to collect data on the occurrence of musculoskeletal symptoms (pain or discomfort) affecting any of the nine body regions (neck, shoulder, upper back, lower back, thigh/hip, knee, wrist/hand, and ankle) in the last seven days. Sick absenteeism was assessed based on the days of sick absenteeism and the frequency of sick absenteeism per month. The outcome variable was measured using two variables: absolute absenteeism and relative absenteeism. Whole and part-workdays missed as a result of personal health reasons in the last month (28 days) were summed, representing absenteeism. Overall, similar data were collected in sections 1 and 2 of the instruments, with the only difference being the follow-up time points.

As for the HPQ, the outcome variable was measured by computing absolute presenteeism and relative presenteeism based on specific questions presented on a scoring scale ranging from 0 = worst job performance to 10 = highest performance [19]. Examples of the questions include “How would you rate your overall job performance on the days you worked during the past four weeks?” The absolute presenteeism score was then computed by multiplying the participants by 10. Hence, the absolute presenteeism score varied from 0 (no performance in work time) to 100 (optimum performance in work time). A similar method was applied for relative presenteeism, based on the difference between the expected number of working hours and the total work hours in the past 4 weeks, divided by the former. All aforementioned time and frequency of outcome assessments were also performed in the control group.

### 2.10. Participants' socio-demographics, medical, and work-related characteristics

A section of the instrument regards participants' socio-demographic characteristics, work-related factors, and medical history. As for socio-demographic characteristics, items are designed to collect information on participants' age, gender, race, medical conditions, monthly income, job category, educational qualification, and marital status. For medical history and health status, the data comprised smoking status, pregnancy status, history of trauma, and surgery. Meanwhile, work-related characteristics entailed working experience, job type (full-time vs. part-time), and engagement in other activities such as hobbies. The final section entailed variables (high, moderate, and low activity) relating to participation in physical activity, defined as the frequency of performing physical activity on an average weekly basis in a calendar year, calculated as MET × minutes/week.

### 2.11. Compliance evaluation and modification

**Figure 1.**
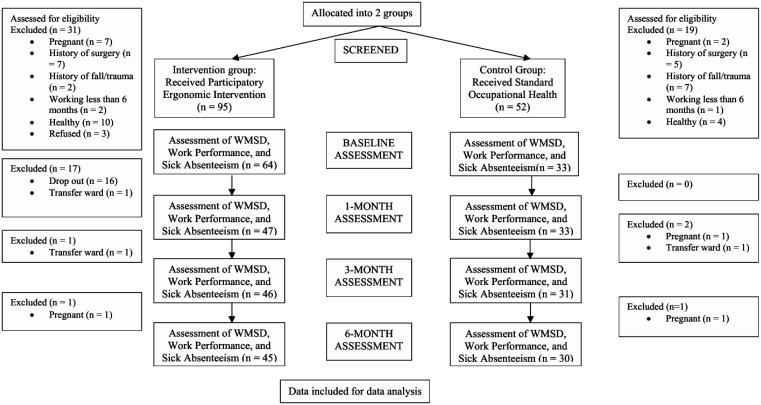
Study flowchart of the cluster quasi-experimental intervention trial.

Participants' compliance was assessed to ensure that nurses complied with the Ergorealm regime contained in the intervention. During the first month post-intervention, a dropout of 50% was observed in the intervention group due to stringent compliance assessment; hence, compliance assessment was amended in the participants' best interest. Specifically, the mandatory monthly compliance assessment and uploading self-recorded PMR video were modified to 3 monthly assessments and optional video uploads. The evaluation of the uploaded videos was mainly to ascertain if the participant was performing the PMR correctly and to assist those experiencing any difficulty. As a result, additional participants were recruited for the intervention group (ward 13U) to compensate for the sample size and assess the intervention's effects. However, the intervention protocol and contents of the PE module remained unchanged. The intervention was administered to these newly recruited participants using the same approach described earlier. Wards 12U and 13U share similar medical ward characteristics and workload and are supervised by the same nursing head (matron). Figure 1 provides further information on the participant recruitment process and follow-up assessments.

### 2.12. Statistical analysis

All statistical analyses were conducted using the Statistical Package for Social Science (SPSS), Version 24 (IBM Corp, Armonk, New York, NY, United States). Dependent variables were recorded as categorical data for the presence or absence of specific WMSD, whereas secondary outcomes (i.e., sick absenteeism scores and work performance scores) were presented as continuous data. Sick absenteeism and work performance scores were checked for normality using the Kolmogorov–Smirnov and Shapiro–Wilks tests. Non-normally distributed data were log-transformed or summarized in median and interquartile ranges, whereas data conforming to normality assumptions were presented in mean and standard deviation (SD).

During the baseline, independent t-test and Mann–Whitney U tests were conducted to ensure that both groups were characteristically similar while identifying potential covariates. All statistical assumptions, either the normality test or the homogeneity of variance, were investigated before conducting the inferential analysis. Missing data analyses were also performed descriptively and compared between the groups to ascertain the extent and identify the appropriate data management approach.

The effectiveness of the intervention module was analyzed using generalized estimating equations (GEEs). First, main group effects were assessed for each outcome between the control and intervention groups. Next, for the comparisons of outcome data between the groups, the first month values were used as the reference group, since the study was designed to determine the intervention's longitudinal effect (i.e., changes in the outcome variables at the third and sixth months relative to the first month). To adjust for the clustered data and small sample corrections, the robust variance estimation method was selected in GEE. The small number of clusters (K = 3) requires a bias correction, the Kauermann and Carroll [23], to the standard sandwich estimator. This correction is critical to maintain accurate Type I error rates and coverage of confidence intervals, as well as improve the GEE model's finite-sample performance. We also used the Mancl and DeRouen [24] estimator in computing the confidence intervals—a process that yields coverage close to the nominal level, especially when the number of clusters is small. In all statistical tests, a p-value of less than 0.05 was considered significant.

## 3. Results

### 3.1. Number of participants recruited and dropout rate

Figure 1 depicts the total number of nurses in the trial and the corresponding dropouts across time. For the intervention wards, during the initial stage, 34 and 30 nurses fulfilling the inclusion criteria were enrolled from 12U and 13U, respectively. One month into the study, half (n = 17) of the participants in 12U dropped out of the trial due to stringent compliance assessment, transfer to another ward, becoming pregnant, and study leave/postgraduate studies. The remaining 17 participants completed the trial in the sixth month post-intervention. Meanwhile, 1 nurse in 13U dropped out of the trial three months after enrollment, followed by another participant in the sixth month. Hence, 28 participants from this ward completed the intervention trial, corresponding to 45 participants in the intervention group.

As for the control group (11U), 52 nurses were screened initially, with 33 fulfilling the inclusion criteria. All participants completed the first month, two dropped out in the third month, and one nurse dropped out in the sixth month. The main reasons for this were transfer to another ward (n = 1) and pregnancy (n = 2). As a result, 30 participants completed the study in the control group.

### 3.2. Participants' demographic characteristics

A total of 75 participants completed the study, with a median age of 26 and 28 years for the control and intervention groups, respectively. Table 2 summarizes the socio-demographic and work-related characteristics of participants in both the intervention and control groups. None of the characteristics were significantly different between the groups, indicating the similarity of participants recruited into the trial. Table 3 presents the participants' health-related characteristics between the intervention and control groups. Excluding physical activities, no other variables were statistically significantly different between the two groups.

**Table 2.** Socio-demographics and work-related characteristics in both intervention and control groups.

Characteristics	Intervention	Control	P-value*

	n (%)	n (%)	
Age (years), median (Q1–Q3)	26.0 (25.0–30.0)	28.0 (24.75–31.25)	0.631
Gender			
Male	6 (13.3)	0 (0.0)	0.075
Female	39 (86.7)	30 (100.0)	
Ethnicity			
Malay	43 (95.6)	29 (96.7)	>0.05
Non-Malay	2 (4.4)	1 (3.3)	
Marital status		
Single	22 (48.9)	19 (63.3)	0.245
Married	23 (51.1)	11 (36.7)	
Highest education level	
Advanced diploma in nursing (post-basic) and above	5 (11.1)	5 (16.7)	0.508
Diploma	40 (88.9)	25 (83.3)	
Monthly gross income	
RM 4000 and below	36 (80.0)	24 (80.0)	>0.05
RM 4001 and above	9 (20.0)	6 (20.0)	
Occupation		
Charge nurse/nurse	43 (95.6)	28 (93.3)	>0.05
Senior sister/matron/sister	2 (4.4)	2 (6.7)	
Job status		
Permanent	39 (86.7)	25 (83.3)	0.746
Contractual	6 (13.3)	5 (16.7)	
Type of working hours	
Office hours/flexible hours	3 (6.7)	2 (6.7)	>0.05
Shift hours	42 (93.3)	28 (93.3)	
Working duration (years), median (Q1–Q3)	4 (2.0–8.0)	5.5 (2.0–9.3)	0.740

Note: *Exact significance (2-sided) is presented for p-value. RM 4000 was used as a cutoff for the monthly gross income because it depicts the average wage/salary of a nurse in Malaysia.

**Table 3.** Distribution of health-related characteristics in the intervention and control groups.

Characteristics	Intervention	Control	p-value*

	n (%)	n (%)	
Physical activity (MET × minutes/week)		
Low	32 (71.1)	12 (40.0)	0.006
Moderate	3 (6.7)	10 (33.3)	
High	10 (22.2)	8 (26.7)	
Hobby			
**Strenuous	5 (11.1)	3 (10.0)	>0.05
Non-strenuous	40 (88.9)	27 (90.0)	
Health status (presence of comorbid)		
Yes	3 (6.7)	4 (13.3)	0.430
No	42 (93.3)	26 (86.7)	
Musculoskeletal symptoms (MSD) Specific body regions		
Neck	32 (71.1)	20 (66.7)	0.799
Shoulder	17 (37.8)	23 (76.7)	0.001
Elbow	4 (8.9)	0 (0.0)	0.145
Wrist/hand	9 (20.0)	11 (36.7)	0.121
Upper back	11 (24.4)	11 (36.7)	0.305
Low back	28 (62.2)	22 (73.3)	0.454
Hip/thigh	4 (8.9)	8 (26.7)	0.055
Knee	6 (13.3)	5 (16.7)	0.746
Ankle/feet	10 (22.2)	12 (40.0)	0.123
Total MSD (number of symptoms), median (Q1–Q3)	2 (1–4)	4 (2–5)	0.033
Sick absenteeism, time (hours), median (Q1–Q3)	
Absolute absenteeism	28 (0–48)	8 (0–37.5)	0.552
Relative absenteeism	0.17 (0–0.25)	0.05 (0–0.20)	0.648
Work performance, performance rate (%), median (Q1–Q3)
Absolute presenteeism	70 (50–80)	80 (70–80)	0.296
Relative presenteeism	1 (0.94–1.00)	1 (0.94–1.14)	0.962

Note: *Exact significance (2-sided) is presented for p-value, comorbid with a known case of diabetes mellitus, hypertension, or dyslipidemia if they are under treatment with anti-diabetics, anti-hypertensives, or lipid-lowering agents. **Strenuous activities refer to physical exertion that makes an individual experience heavy and rapid breathing and the inability to continue speaking without pausing for breath. It is characterized by the aforementioned physiological responses and physical demand that stem from activities such as lifting heavy weights, repetitive arm movements, awkward postures, or prolonged standing.

The number of participants reporting neck pain (71.1% and 66.7%), wrist pain (20.0% and 36.7%), upper back pain (24.4% and 36.7%), low back pain (62.2% and 73.3%), knee pain (13.3% and 16.7%) and ankle/feet pain (22.2% and 40.0%) in the intervention and control group were not significantly different (P > 0.05). Most participants in both groups did not report elbow pain during the baseline assessment, and the p-value was not significant (P = 0.145). However, a significantly higher number of participants in the control group complained of shoulder pain (37.8% and 76.7%, P = 0.001) and hip/thigh pain (8.9% and 26.7%, P = 0.055) relative to the intervention group.

In terms of absolute sick absenteeism at baseline, the intervention group reported a median of 28 hours (0–48) at baseline, while the control group recorded a median of 8 hours (0–37.5) with no statistically significant differences (P = 0.552). For relative absenteeism at baseline, the intervention group reported a median of 0.17 (0–0.25) compared to the control group at 0.05 (0–0.20) with no statistically significant differences (P = 0.648). For baseline work performance, the intervention and control groups recorded a median absolute presenteeism of 70% (50–80) and 80% (70–80), respectively, with no statistically significant differences (P = 0.296). Relative presenteeism in the intervention group at baseline was similar in both groups (i.e., 100%) with no statistical significance (P = 0.962).

### 3.3. Effects of participatory ergonomic intervention on work-related musculoskeletal disorders

As shown in the final models (Model 1 = adjusted OR with physical activity, and Model 2 = adjusted OR with physical activity and baseline symptoms), the intervention effect was statistically significant in reducing neck (Model 1: AOR = 0.43, 95% CI 0.21–0.90; Model 2: AOR = 0.43, 95% CI 0.21–0.90) and low back symptoms (Model 1: AOR = 0.46, 95% CI 0.22–0.97; Model 2: 0.45, 95% CI 0.21–0.97). Similar results were obtained across time periods with the first month as a reference point, evidenced by a significant reduction in neck pain (AOR = 0.36, 95% CI 0.21–0.62) and low back pain (AOR = 0.29, 95% CI 0.15–0.56) during the third month (Table 4). However, there was no significant difference in the odds of upper back pain between the groups in the unadjusted and adjusted models, suggesting that the intervention module was effective in addressing neck and low back pain with minimal impact on the upper back.

**Table 4.** Association between Ergorealm and WMSD for various body regions.

Parameters	Crude OR		Model 1: adjusted OR		Model 2: adjusted OR	
	OR (95% CI)	p-value	OR (95% CI)	p-value	OR (95% CI)	p-value
Neck
Group						
Control	1		1		1	
Intervention	0.51 (0.26–1.00)	0.048	0.43 (0.21–0.90)	0.024	0.42 (0.21–0.87)	0.020
Time						
First month	1		1		1	
Third month	0.33 (0.14–0.78)	0.012	0.32 (0.13–0.78)	0.012	0.31 (0.13–0.78)	0.012
Sixth month	0.36 (0.21–0.62)	<0.001	0.36 (0.21–0.61)	<0.001	0.34 (0.20–0.59)	<0.001
Upper back
Group						
Control	1		1		1	
Intervention	0.88 (0.30–2.58)	0.815	0.872 (0.20–3.77)	0.855	0.88 (0.23–3.42)	0.853
Time						
First month	1		1		1	
Third month	0.37 (0.12–1.17)	0.090	0.36 (0.11–1.17)	0.090	0.36 (0.11–1.17)	0.090
Sixth month	0.67 (0.28–1.60)	0.366	0.67 (0.28–1.61)	0.364	0.67 (0.28–1.61)	0.364
Low back
Group						
Control	1		1		1	
Intervention	0.50 (0.25–1.00)	0.048	0.46 (0.22–0.97)	0.042	0.45 (0.21–0.97)	0.041
Time						
First month	1		1		1	
Third month	0.49 (0.24–0.98)	0.045	0.49 (0.24–0.98)	0.045	0.49 (0.24–0.98)	0.044
Sixth month	0.29 (0.15–0.56)	<0.001	0.29 (0.15–0.56)	<0.001	0.29 (0.15–0.56)	<0.001
Shoulder
Control	1		1		1	
Intervention	0.41 (0.19–0.87)	0.020	0.33 (0.14–0.78)	0.012	0.38 (0.17–0.88)	0.024
Time						
First month	1		1		1	
Third month	0.46 (0.21–1.03)	0.060	0.44 (0.18–1.04)	0.061	0.44 (0.18–1.04)	0.061
Sixth month	0.36 (0.16–0.83)	0.015	0.34 (0.14–0.81)	0.015	0.34 (0.14–0.81)	0.015
Elbow
Group						
Control	1		1		n/a	n/a
Intervention	0.58 (0.08–4.08)	0.580	0.56 (0.05–6.84)	0.649	n/a	n/a
Time						
First month	1		1		n/a	n/a
Third month	0.49 (0.04–5.67)	0.571	0.49 (0.04–5.66)	0.57	n/a	n/a
Sixth month	1.00 (0.13–7.54)	0.998	1.00 (0.13–7.57)	0.997	n/a	n/a
Wrist/hand
Group						
Control	1		1		1	
Intervention	0.48 (0.16–1.44)	0.192	0.41 (0.11–1.53)	0.185	0.49 (0.12–1.91)	0.301
Time						
First month	1		1		1	
Third month	0.55 (0.14–2.06)	0.371	0.54 (0.14–2.07)	0.371	0.54 (0.14–2.10)	0.375
Sixth month	1.00 (0.41–2.42)	>0.05	1.00 (0.41–2.43)	>0.05	1.00 (0.41–2.45)	0.996
Knees
Control	1		1		1	
Intervention	0.20 (0.05–0.79)	0.021	0.15 (0.04–0.61)	0.008	0.13 (0.04–0.50)	0.003
Time						
First month	1		1		1	
Third month	0.57 (0.12–2.70)	0.481	0.56 (0.12–2.76)	0.480	0.54 (0.09–3.18)	0.491
Sixth month	1.00 (0.34–2.91)	0.999	1.00 (0.33–3.00)	0.999	0.99 (0.30–3.31)	0.989
Ankles/feet
Group						
Control	1		1		1	
Intervention	0.34 (0.12–0.94)	0.038	0.26 (0.09–0.71)	0.009	0.27 (0.10–0.73)	0.010
Time						
First month	1		1		1	
Third month	0.69 (0.19–2.46)	0.566	0.68 (0.19–2.49)	0.565	0.68 (0.19–2.52)	0.568
Sixth month	0.69 (0.24–1.94)	0.480	0.68 (0.24–1.96)	0.479	0.68 (0.24–1.97)	0.478

Note: *Exact significance (2-sided) is presented for p-value. Model 1: Adjusted OR (with physical activity). Model 2: Adjusted OR (with physical activity and baseline symptoms).

The intervention effect was also statistically significant in reducing shoulder pain (Model 1: AOR = 0.33, 95% CI 0.14–0.78; Model 2: AOR = 0.38, 95% CI 0.17–0.88) compared to the control group. This effect was significant across time, evidenced by a reduction in shoulder pain symptoms (AOR = 0.34, 95% CI 0.14–0.81) during the sixth month relative to the first month post-intervention. In contrast, no statistically significant reduction of symptoms was observed for the elbow and wrist/hand regions, as shown in the unadjusted and adjusted models. The adjusted models also depicted significantly lower odds of WMSD affecting the knees (Model 1: AOR = 0.15, 95% CI 0.04–0.61; Model 2: AOR = 0.13, 95% CI 0.04–0.50) and ankles/feet (Model 1: AOR = 0.26, 95% CI 0.09–0.71; Model 2: AOR = 0.27, 95% CI 0.10–0.73) in the intervention group. However, the intervention effect was not statistically significant across time.

### 3.4. Effects of participatory ergonomic intervention on sick absenteeism

In the adjusted models with physical activity, no statistically significant effect was observed in absolute absenteeism (β = −0.40, 95% CI −0.86, −0.07, P = 0.092) between the intervention and control groups (Supplementary Table S1). However, the intervention group experienced a significant reduction in relative absenteeism compared to the control group upon adjusting for physical activity alone (β = −0.36, 95% CI −0.67, −0.05, P = 0.025) and factoring together physical activity and baseline symptoms (β = −0.41, 95% CI −0.76, −0.06, P = 0.023). Upon considering the time and follow-up points, the adjusted model revealed no statistical difference in absolute absenteeism (β = 0.32, 95% CI −0.26, 0.90, P = 0.277) and relative absenteeism (β = 0.34, 95% CI −0.19, 0.86, P = 0.210) either at the third month or sixth month compared to the first month post-intervention.

### 3.5. Effects of participatory ergonomic intervention on work performance

The intervention group showed no significant difference (P > 0.05) in absolute presenteeism (adjusted Model 1: β = 0.05, 95% CI −0.04, 0.14; adjusted Model 2: β = 0.07, 95% CI −0.01, 0.15). In contrast, relative presenteeism was statistically higher in the intervention group compared to the control group, as evidenced in adjusted models (Model 1: AOR = 0.11, 95% CI 0.01, 0.20, P = 0.024; Model 2: β = 0.10, 95% CI 0.01, 0.19, P = 0.033). Overall, the intervention had a small positive effect on absolute presenteeism but a much greater and significant effect on relative presenteeism as demonstrated in both crude and adjusted analyses (Supplementary Table S2). Only a group effect was observed, as no statistical difference was detected in any of the work performance variables upon considering the follow-up periods (lack of time effect).

## 4. Discussion

### 4.1. General discussion (implementation and compliance with the intervention)

The primary objectives of this study were to assess the effects of a participatory ergonomic intervention on work performance, sick absenteeism, and WMSDs among nurses. The control and intervention groups were characteristically similar for most of the demographic factors recorded during recruitment, except for gender and working experience, whereby higher proportions of male and less experienced nurses were present in the intervention relative to the control groups. While working experience may not have a substantial effect on the trial results, gender is a critical factor given that females are more likely to experience pain because of their relatively lower upper body strength compared to males [25].

The initial stringent compliance assessment led to a high dropout rate during the first month post-intervention, which was then addressed by modifying the compliance assessment. Thereafter, most nurses accepted and tolerated the intervention as evidenced by participants' compliance rate, which was positive, especially from the second week until the third month. The positive outcomes might have been influenced by improved knowledge of ergonomic training and PMR, perceived benefits of the program, and a low number of complaints. This was also evidenced in the low dropout rates after the first month of the trial until completion. Prior studies have shown that participants in ergonomic interventions are more likely to remain in a clinical trial once they become familiar with the intervention contents and experience the positive benefits [13],[26]. Hence, given the comprehensive contents of the ergonomic training manual developed in this study, nurses in the intervention group might have become more aware of ergonomic factors contributing to WMSD and gained better knowledge of using assisted equipment and devices for patient handling and transfers. These events have positive impacts on compliance and practical rates. Therefore, a participatory approach is indeed required in a nursing setting, clarifying the importance of tailoring the ergonomic training structure to individually adapted exercises [26].

### 4.2. Prevalence of WMSDs

In this study, more than 70% of nurses experienced various types of WMSD, predominantly affecting the neck, low back, and shoulders. These findings corroborate reports from studies conducted in Malaysia and other countries, with WMSD constituting a major driver of occupation injuries, disability, and production losses [4],[7],[8],[27]. Nevertheless, a high risk or prevalence of WMSD may not occur concurrently with the associated secondary outcomes, such as sick absenteeism and work performance [7],[13],[15]. This stems from the fact that sick leaves and work performance are also multifactorial events, indicating the need for an interaction between WMSD and other relevant factors. As observed in the present study, despite the high prevalence of WMSD at baseline in both groups, the participants did not report significant levels of sick absenteeism and low work performance, results that mirror reports from prior studies [7],[15].

### 4.3. Effects of the intervention on the primary outcomes

#### 4.3.1. Work-related musculoskeletal disorders

Overall, the intervention was effective in addressing three primary MSDs, namely neck pain, shoulder pain, and low back pain. The findings revealed that ergonomic training facilitated a reduction in neck pain, which was sustainable from the first to the sixth month post-intervention. Aligning with this result, Shariat et al. [28] reported a significant reduction in neck pain following ergonomic intervention, whereas the control group experienced persistent neck discomfort. Cote et al. [29] also found improvement in neck symptoms after workstation adjustments, but additional treatments such as physical fitness were necessary. A factor that may explain the significant decline in neck pain is the upright posture when handling patients and the support of nurses' arms on the table when sitting in their offices. This support reduces activity in the upper deltoid and trapezius muscles and diminishes shoulder and neck torque due to lower biomechanical load [30],[31]. These events were further complemented with the PMR exercise, which helps in relaxing the muscles around the neck, shoulder, upper, and lower back.

The intervention group also experienced significantly lower odds of shoulder pain in the sixth month compared to the control group, which can be linked to the positive impacts of workstation adjustments in reducing or preventing the development of musculoskeletal pain [32]. The ergonomic training emphasized the use of lumbar support and seat adjustments, as well as assisted patient lifting devices. Previous studies have reported a reduction in lower back pain after workplace adjustments, which facilitated the adoption of neutral positions [28],[33]. As found in the present study setting, lumbar support was sparingly used by nurses, and most of the recruited nurses were not well-informed about proper workplace ergonomics prior to this study. As for the other body regions, including the knees and ankles/feet, the lack of positive results may be linked to the low reports and low pain intensity for the body regions in both groups.

#### 4.3.2. Sick absenteeism and work performance

No statistical difference was observed between the intervention and control groups in terms of sick absenteeism and work performance post-intervention, except for relative absenteeism and relative presenteeism as evidenced in adjusted analyses. While some studies have shown that participatory ergonomics resulted in reducing the risks of WMSD, occupational injuries, compensation claims, work absenteeism, and productivity loss [12],[34],[35], others found no significant difference in these outcome measures between intervention and control groups [36].

Diverse factors, ranging from the contents of the intervention, duration, adherence, nature of the work, workplace environment, to the method of administering the intervention, may contribute to the discrepancies in results. As observed in the present study, adjustment for physical activities and baseline symptoms revealed that the intervention group experienced a significant reduction in relative absenteeism and higher scores for relative presenteeism compared to the control group. Although these factors might contribute to masking the intervention's main effects in addressing relative absenteeism and presenteeism, it remains uncertain if such effects can translate to remarkable changes in overall sick leave and work performance. Further insight into the findings was gleaned from the comparison of sick absenteeism and work performance measures across time in both groups. Therefore, evidence from this study depicts that the PE intervention module was more effective in addressing pertinent WMSD, such as shoulder, neck, and low back pain, but the effects were insufficient to exert a significant change in participants' sick absenteeism and work performance.

The present findings regarding sick absenteeism and work performance are comparable to prior studies. Blangsted et al. [37] found that absenteeism and work performance remained unchanged after a 12-month workplace shoulder and neck exercise training. Meanwhile, Justesen et al. [38] reported improved levels of absenteeism and presenteeism following a one-year workplace individualized physical exercise training of adequate adherence. The main difference between these aforementioned studies and the present one is the nature and duration of the intervention. This study deployed a shorter ergonomic program of 24 weeks (6 months), with a continuation of the regimen facilitated by distributing well-designed manuals and materials for independent activity. In addition, only post-intervention impacts were investigated as the primary objectives, in contrast to prior research exploring longer-term effects [38]. The recommendation of Pereira et al. [39] was also followed in this study by using multidimensional workplace interventions involving exercise and workplace ergonomics, thereby increasing the opportunity to augment nurses' productivity. Considering that a minimum of 10 weeks is recommended to ascertain the effects of workplace interventions for MSD [26], it was not surprising that no significant results were detected for most MSD during the first month of the intervention. Nevertheless, the lack of significant findings between the groups at third- and sixth-month post-intervention requires further investigation.

Previous work with similar results to the present study argued that HPQ's 28-day recall may influence participants' reporting post-intervention performance levels that do not reflect the maximum benefit of the intervention. Another important point is the low levels of absenteeism among the nurses in both groups during the recruitment, thereby limiting the chance of obtaining a significant effect post-intervention. The roles of confounding factors that were not accounted for, given the sampling criteria and study population, may also contribute to the present findings. For instance, a similar study conducted in Australia, whereby ergonomic training had no significant effects on work performance and sick absenteeism, suggested that intervention delivery scheduling might have been affected by unmeasured socio-economic disparities and seasonal variation [40]. The potential influence of such events on the results is low, given that most socio-economic factors and demographic characteristics were similar in both groups.

Available evidence from this study reflects that a combination of workplace ergonomics and PMR exercise is effective in addressing important WMSD among nurses, but whether the intervention translates to better work performance and lower levels of absenteeism remains unclear. Multifaceted approaches are indeed required to ensure changes in practice and to enhance safety culture, including workflow processes, skills, ongoing training, and interpersonal communication about risk [11],[41],[42]. Although these intrinsic events were not investigated in this study, ergonomic approaches and guidelines provided in the training module might have improved nurses' knowledge and skills in patient handling, understanding the risks and consequences of awkward postures, thereby modifying behavioral change to engage in safety practices [26]. Our results on WMSDs support recent arguments that multiple interventions are emerging as the most effective strategies for preventing these health problems among nurses [43],[44].

The results from this study reflect the impact of ergonomic training and PMR in reducing the risk of WMSD, reinforcing general recommendations for the development of a safety culture in healthcare institutions, particularly among nurses. The training content was successfully adapted to the nursing field as a promising module for ameliorating WMSD in nurses, but the available data are insufficient to ascertain its impacts on work performance and sick absenteeism.

### 4.4. Study limitations

There are important methodological limitations in this study. Since this study was a single-center trial, it has limited external validity. We used a single tertiary hospital in Malaysia due to the challenge in identifying participants fulfilling the eligibility criteria and the resource intensity associated with intervention implementation and follow-up assessments. While this sampling method assisted the researcher in having more control over the participants to be selected and recruiting nurses with similar characteristics, the results might not be directly generalized to other nursing operations in Malaysia and other countries. For instance, working conditions are expected to differ between public and private healthcare centers, which will definitely influence the risk of WMSD, work performance, and sick absenteeism. Information on the intervention costs and resource intensity is also crucial in ascertaining the realistic implementation and sustainability of the intervention.

The HPQ and Nordic musculoskeletal questionnaires are self-reporting instruments, which are subjective and vulnerable to recall bias. Although these instruments have been validated among nurses and found to be reliable, using the HPQ's 28-day recall in measuring participants' work performance may not fully reflect the actual benefit of the intervention. In addition, we did not collect data on the severity of WMSDs since there is no provision for the variable in the short NMQ version. Although our objective was to assess self-reported presence or absence of WMSD at specific body regions, the extended version can be used in future studies to reduce the subjectivity associated with self-reporting instruments and to monitor musculoskeletal conditions more objectively. As for the ergonomic training module, the contents were synthesized based on the results from a detailed literature review and FGD, indicating the strengths of the research approach [13],[45]. However, no blinding approach was implemented since it was unrealistic to ensure that participants were unaware of the groups they were allocated to. Moreover, the principal investigator conducted participant recruitment, whereas self-reporting instruments were used for the outcome assessment, making it challenging to perform either a single or double-blinded trial.

Attempts were made in the intervention to integrate components addressing both psychosocial and organizational risk factors for WMSD. Examples include educational content on health promotion activities, workstation adjustment, and usage of patient handling devices; these approaches were limited to ergonomic education and interaction with the nurses during the FGD and supported with videos. In other words, we did not introduce any new patient handling device or workstation but rather educated and encouraged nurses to use existing equipment.

The number of clusters in this study was relatively low, with 45 and 30 participants completing the trial in the intervention and control groups, respectively. Despite using small-sample correction procedures in GEE and providing insights into ergonomic approaches for addressing WMSD in a large tertiary hospital, the number of clusters affects the generalizability of the findings. Future research requires cluster-randomized designs with adequate clusters, longer follow-up, and hybrid interventions integrating ergonomic, organizational, and psychosocial components.

## 5. Conclusion

This cluster quasi-experimental study revealed the effectiveness of the newly developed ergonomic training module in addressing WMSD, sick absenteeism, and work performance among ward nurses. Overall, the results are promising as the intervention group demonstrated significantly lower odds of common WMSDs affecting the neck, low back, shoulder, knee, and ankle during the follow-up periods compared to the control group. However, improved musculoskeletal health observed in the intervention group was not sufficient to facilitate significant changes in self-reported sick absenteeism and work performance. In conclusion, while the intervention was effective in addressing important WMSD among nurses, it remains unclear if the newly developed ergonomic training program would translate to better work performance and lower levels of sick absenteeism. These findings highlight the need to establish a safety culture among nursing professionals and opportunities to explore possible adjustments in the ergonomic training module for better outcomes and application to a wider population of nurses.

## Use of AI tools declaration

The authors declare they have not used Artificial Intelligence (AI) tools in the creation of this article.


