# Supplementary material for: Development and evaluation of a participatory ergonomic intervention for the reduction of work-related musculoskeletal disorders among nurses
Source: AIMS Public Health. 2025 Dec 22;12(4):1240–64. doi: 10.3934/publichealth.2025063 (PMC12795764; doi:10.3934/publichealth.2025063)
Supplement: Supplementary file 1 [file publichealth-12-04-063-s001.pdf]

Public Health

AIMS Public Health, 12 (4): 1240–1264.
DOI: 10.3934/publichealth.2025063
Received: 26 September 2025
Revised: 01 December 2025
Accepted: 16 December 2025
Published: 22 December 2025

https://www.aimspress.com/journal/aimsph

Research article

Development and evaluation of a participatory ergonomic intervention for the reduction of work-related musculoskeletal disorders among nurses

Guganesan Krishnanmoorthy[1], Rama Krishna[1,2], Faiz Baharudin[3,4], Mohd Amirul Asraf Shah Nizamuddin[5], Nik Hasmumthaj Nik Hassan[6], Mahmoud Danaee[1], Victor C W Hoe[1,7] and Sanjay Rampal[1,7,*]

[1] Department of Social and Preventive Medicine, Faculty of Medicine, Universiti Malaya, Kuala Lumpur, Malaysia
[2] Occupational Health Clinic, Universiti Malaya Medical Centre (UMMC), Kuala Lumpur, Malaysia
[3] Occupational Safety, Health and Environment Centre, Universiti Malaya, Kuala Lumpur, Malaysia
[4] Occupational Safety, Health and Environment Unit, Universiti Malaya Medical Centre, Kuala Lumpur, Malaysia
[5] Department of Civil, Environmental and Mining Engineering, University of Western Australia, Western Australia, Australia
[6] Department of Nursing, Universiti Malaya Medical Centre (UMMC), Kuala Lumpur, Malaysia.
[7] Centre for Epidemiology and Evidence-based Practice, Department of Social and Preventive Medicine, Faculty of Medicine, Universiti Malaya, Kuala Lumpur, Malaysia

* **Correspondence:** Email: srampal@um.edu.my; Tel: +60379677351.

Table S1. Association between ergorealm and sick absenteeism.

Parameters	Crude					Model 1: Adjusted with physical activity					Model 2: Adjusted with physical activity and baseline				
	β	SE	95% CI		p-value	β	SE	95% CI		p-value	β	SE	95% CI		p-value
			LL	UL				LL	UL				LL	UL	
Absolute absenteeism															
Group															
Control	1					1					1				
Intervention	−0.81	0.501	−1.79	0.17	0.107	−0.40	0.235	−0.86	0.07	0.092	−0.37	0.200	−0.76	0.02	0.064
Time															
First month	1					1					1				
Third month	0.27	0.323	−0.37	0.90	0.407	0.32	0.295	−0.26	0.90	0.277	0.39	0.212	−0.03	0.81	0.066
Sixth month	0.66	0.221	−0.37	0.50	0.765	0.14	0.215	−0.29	0.56	0.530	0.17	0.180	−0.19	0.52	0.356
Relative absenteeism															
Group															
Control	1					1					1				
Intervention	−0.26	0.198	−0.65	0.13	0.194	−0.36	0.159	−0.67	−0.05	0.025	−0.41	0.179	−0.76	−0.06	0.023
Time															
First month	1														
Third month	0.28	0.255	−0.22	0.78	0.267	0.34	0.268	−0.19	0.86	0.210	0.29	0.233	−0.17	0.74	0.216
Sixth month	0.21	0.171	−0.12	0.55	0.215	0.25	0.187	−0.12	0.61	0.187	0.17	0.175	−0.17	0.51	0.336

Note: *Exact significance (2 sided) was presented for p-value, LL = lower limit, UL = upper limit, CI = confidence interval, β = standardized coefficient, SE = standard error. Model 1: Adjusted OR (With physical activity). Model 2: Adjusted OR (With physical activity and baseline symptoms)

Table S2. Association between ergorealm and work performance.

Parameters	Crude					Model 1: Adjusted OR					Model 2: Adjusted OR				
	OR	SE	95% CI		p-value	OR	SE	95% CI		p-value	OR	SE	95% CI		p-value
			LL	UL				LL	UL				LL	UL	
Absolute presenteeism															
Group															
Control	1					1					1				
Intervention	0.06	0.044	−0.02	0.15	0.162	0.05	0.045	−0.04	0.14	0.284	0.07	0.040	−0.01	0.15	0.075
Time															
First month	1					1					1				
Third month	−0.01	0.055	−0.11	0.10	0.909	0.00	0.054	−0.10	0.11	0.971	0.01	0.054	−0.10	0.11	0.915
Sixth month	−0.01	0.038	−0.09	0.06	0.778	−0.01	0.038	−0.08	0.07	0.875	−0.01	0.036	−0.08	0.07	0.868
Relative presenteeism															
Group															
Control	1					1					1				
Intervention	0.11	0.048	0.020	0.208	0.017	0.11	0.048	0.014	0.202	0.024	0.10	0.047	0.01	0.19	0.033
Time															
First month											1				
Third month	−0.01	0.086	−0.17	0.16	0.942	−0.01	0.087	−0.18	0.17	0.955	−0.01	0.087	−0.18	0.16	0.937
Sixth month	−0.06	0.042	−0.14	0.03	0.182	−0.05	0.042	−0.14	0.03	0.203	−0.04	0.041	−0.12	0.04	0.302

Note: *Exact significance (2 sided) was presented for p-value. Model 1: Adjusted OR (With physical activity). Model 2: Adjusted OR (With physical activity and baseline symptoms)

AIMS Press

© 2025 the Author(s), licensee AIMS Press. This is an open access article distributed under the terms of the Creative Commons Attribution License (https://creativecommons.org/licenses/by/4.0)
